# Point of Care Ultrasound Literature Primer: Key Papers on Focused Assessment With Sonography in Trauma (FAST) and Extended FAST

**DOI:** 10.7759/cureus.30001

**Published:** 2022-10-06

**Authors:** Daniel J Kim, Colin Bell, Tomislav Jelic, Gillian Sheppard, Laurie Robichaud, Talia Burwash-Brennan, Jordan Chenkin, Elizabeth Lalande, Ian Buchanan, Paul Atkinson, Rajiv Thavanathan, Claire Heslop, Frank Myslik, David Lewis

**Affiliations:** 1 Department of Emergency Medicine, University of British Columbia, Vancouver, CAN; 2 Department of Emergency Medicine, Vancouver General Hospital, Vancouver, CAN; 3 Department of Emergency Medicine, University of Calgary, Calgary, CAN; 4 Department of Emergency Medicine, University of Manitoba, Winnipeg, CAN; 5 Discipline of Emergency Medicine, Memorial University of Newfoundland, St. John's, CAN; 6 Department of Emergency Medicine, McGill University, Montreal, CAN; 7 Department of Family and Emergency Medicine, Université de Montréal, Montreal, CAN; 8 Division of Emergency Medicine, Department of Medicine, University of Toronto, Toronto, CAN; 9 Department of Emergency Medicine, Université Laval, Quebec City, CAN; 10 Department of Emergency Medicine, McMaster University, Hamilton, CAN; 11 Department of Emergency Medicine, Saint John Regional Hospital, Saint John, CAN; 12 Department of Emergency Medicine, Dalhousie Medicine New Brunswick, Saint John, CAN; 13 Department of Emergency Medicine, University of Ottawa, Ottawa, CAN; 14 Division of Emergency Medicine, Department of Medicine, University of Western Ontario, London, CAN

**Keywords:** point of care ultrasound, pneumothorax (ptx), free fluid, e-fast, fast, trauma, ultrasound

## Abstract

Objective

The objective of this study is to identify the top five most influential papers published on focused assessment with sonography in trauma (FAST) and the top five most influential papers on the extended FAST (E-FAST) in adult patients.

Methods

An expert panel was recruited from the Canadian Association of Emergency Physicians (CAEP) Emergency Ultrasound Committee and the Canadian Ultrasound Fellowship Collaborative. These experts are ultrasound fellowship-trained or equivalent, are involved with point-of-care ultrasound (POCUS) research and scholarship, and are leaders in both the POCUS program at their local site and within the national Canadian POCUS community. This 14-member expert group used a modified Delphi process consisting of three rounds of sequential surveys and discussion to achieve consensus on the top five most influential papers for FAST and E-FAST.

Results

The expert panel identified 56 relevant papers on FAST and 40 relevant papers on E-FAST. After completing all three rounds of the modified Delphi process, the authors identified the top five most influential papers on FAST and the top five most influential papers on E-FAST.

Conclusion

We have developed a reading list of the top five influential papers for FAST and E-FAST that will benefit residents, fellows, and clinicians who are interested in using POCUS in an evidence-informed manner.

## Introduction

The use of point-of-care ultrasound (POCUS) was first described in the emergency medicine (EM) literature in 1988 [1]. The American College of Emergency Physicians (ACEP) published its first statement endorsing the use of ultrasound by appropriately trained physicians in 1990 [2], and the Canadian Association of Emergency Physicians (CAEP) issued its first position statement on ultrasound in 1999 supporting its availability in the emergency department (ED) 24 hours per day and its use by emergency physicians [3]. EM has taken the lead on integrating POCUS into residency training and clinical use [3,4], but more recently, a broad range of specialties have also adopted its use, including critical care, internal medicine, anesthesia, hospital medicine, and pediatrics [5].

POCUS growth as a subspecialty has been dramatic, as it has been implemented in educational requirements, practice guidelines, clinical privileging, and research. The evidence base supporting the use of POCUS is continuously growing and becoming more robust [6-8]. While many important POCUS papers have been published, there have been few systematic attempts to identify the most influential papers in this field [6].

The objective of this series is to systematically generate a list of the most influential POCUS papers published for each major application or use of POCUS. Such a list will be useful as an educational resource for residents and fellows and as a literature repository for evidence-informed practicing clinicians in all specialties. It will also be useful to researchers who are interested in improving the methodological soundness of the POCUS literature base. The objective of this study is to use a modified Delphi process to identify the top five most influential papers published on focused assessment with sonography in trauma (FAST) and the top five most influential papers published on the extended FAST (E-FAST) in adult patients.

## Materials and methods

Study design

This was a modified Delphi process [9,10] using sequential surveys and discussion amongst the expert group to build consensus and identify the most influential papers on FAST and E-FAST. The University of Manitoba Office of Research Ethics and Compliance confirmed that research ethics was not required for this process. Our intention is to perform a similar process for each major application or use of POCUS and to create a series of such articles.

Participants

The expert panel is a group of 14 participants in the field of POCUS who were recruited from the CAEP Emergency Ultrasound Committee and the Canadian Ultrasound Fellowship Collaborative. These experts are ultrasound fellowship trained or equivalent, involved with POCUS research and scholarship, and leaders in the POCUS program at their local site and within the national Canadian POCUS community. These individuals were invited by email to participate in the modified Delphi process.

Modified Delphi process

Our modified Delphi process involved three rounds of surveys using Qualtrics (Qualtrics International, Seattle, WA) distributed by email, where participants were reminded by up to a maximum of two emails at weekly intervals to complete each round’s survey. The rounds were distributed in three-week intervals starting in April 2022. Individual submissions were anonymized, but the results of each round of voting were distributed to all of the members of the expert panel.

In round one, participants were asked to nominate five to 10 papers they considered the most influential for FAST (ultrasound for assessment of trauma and injury to the chest, abdomen, or pelvis, including cardiac, intra-abdominal, and pelvic injury, but excluding pneumothorax and hemothorax) and five to 10 papers for E-FAST (ultrasound for traumatic pneumothorax and hemothorax). The term “influential” is defined as important in informing practitioners on how to use POCUS in clinical practice, and interpretation of this term was left to the individual with the anticipation that the expert panel would ultimately achieve consensus after the modified Delphi process. There were no exclusions based on publication date, type of study, or language of the paper, but any studies enrolling only pediatric patients were excluded.

The results of round one were used to create the survey instrument for round two. In round two, participants were asked to select their top five papers for FAST and top five papers for E-FAST. The papers that appeared on the participant’s lists were then collated in order of the most to least number of votes. Papers not receiving any votes were subsequently excluded. Papers receiving two or fewer votes were also excluded, but members of the expert panel were allowed to advocate for the inclusion of a maximum of one of these papers per panel member for inclusion in round three. The expert panel met in person at the CAEP 2022 Annual Conference in Quebec City, Canada, on May 29, 2022 to discuss the results of round two.

For round three, this collated list was sent to the participants, and participants were again asked to select their top five papers for FAST and top five papers for E-FAST. In addition, they were asked to rank their top five selections in order, from most influential to least influential. The paper rated most influential was given a score of five, the next most influential paper was given a score of four, and so forth until the least influential paper was given a score of one. These scores were tallied together, and the five highest scoring papers for FAST and the five highest scoring papers for E-FAST were identified as the most influential papers for these POCUS applications.

## Results

A total of 14 expert panel members participated in all three rounds of the modified Delphi process. The Appendix lists the members of the panel and their academic affiliations. After round one, a total of 59 papers were nominated for FAST, but three pediatric studies were excluded. Therefore, 56 papers were included in round two. For E-FAST, 47 papers were nominated, but seven were excluded as they did not study the use of POCUS for traumatic pneumothorax or hemothorax; 40 papers were included in round two. After round two, there were 12 candidate papers for FAST and 13 candidate papers for E-FAST in round three. Ultimately, the three-round voting process allowed us to generate a rank order list of these papers in order of most to least influential in tables one and two.

**Table 1:** All FAST papers eligible for round three of the Delphi process, along with their votes in round two and votes and total score in round three. Top five papers are indicated in "final rank" column. FAST: Focused assessment with sonography in trauma

Paper	Round Two Votes for Top Five (No (%))	Round Three Votes for Top Five (No (%))	Round Three Total Score	Final Rank
Melniker LA et al. Randomized controlled clinical trial of point-of-care, limited ultrasonography for trauma in the emergency department: the first sonography outcomes assessment program trial. Ann Emerg Med 2006;48(3):227-35 [11]	9 (64%)	13 (93%)	56	1
Inaba K et al. FAST ultrasound examination as a predictor of outcomes after resuscitative thoracotomy: a prospective evaluation. Ann Surg 2015;262(3):512-8 [12]	7 (50%)	10 (71%)	28	2
Ma OJ et al. Prospective analysis of a rapid trauma ultrasound examination performed by emergency physicians. J Trauma 1995;38(6):879-85 [13]	3 (21%)	8 (57%)	24	3
Plummer D et al. Emergency department echocardiography improves outcome in penetrating cardiac injury. Ann Emerg Med 1992;21(6):709-12 [14]	4 (29%)	7 (50%)	21	4
Stengel D et al. Point-of-care ultrasonography for diagnosing thoracoabdominal injuries in patients with blunt trauma. Cochrane Database Syst Rev 2018;12(12):CD012669 [15]	7 (50%)	7 (50%)	19	5
Lobo V et al. Caudal Edge of the Liver in the Right Upper Quadrant (RUQ) View Is the Most Sensitive Area for Free Fluid on the FAST Exam. West J Emerg Med 2017;18(2):270-280 [16]	4 (29%)	7 (50%)	13	
Netherton S et al. Diagnostic accuracy of eFAST in the trauma patient: a systematic review and meta-analysis. CJEM 2019;21(6):727-738 [17]	4 (29%)	5 (36%)	12	
Rozycki GS et al. Early detection of hemoperitoneum by ultrasound examination of the right upper quadrant: a multicenter study. J Trauma 1998;45(5):878-83 [18]	3 (21%)	3 (21%)	11	
Boulanger BR et al. Prospective evidence of the superiority of a sonography-based algorithm in the assessment of blunt abdominal injury. J Trauma 1999;47(4):632-7 [19]	3 (21%)	4 (29%)	9	
Long B, April MD. What Is the Diagnostic Accuracy of Point-of-Care Ultrasonography in Patients With Suspected Blunt Thoracoabdominal Trauma? Ann Emerg Med 2019;74(3):400-402 [20]	3 (21%)	3 (21%)	9	
Lalande E et al. Is point-of-care ultrasound a reliable predictor of outcome during traumatic cardiac arrest? A systematic review and meta-analysis from the SHoC investigators. Resuscitation 2021;167:128-136 [21]	2 (14%)	2 (14%)	7	
Nishijima DK. Does this adult patient have a blunt intra-abdominal injury? JAMA 2012;307(14):1517-27 [22]	2 (14%)	1 (7%)	1	

**Table 2:** All E-FAST papers eligible for round three of the Delphi process, along with their votes in round two and votes and total score in round three. Top five papers are indicated in "final rank" column. E-FAST: Extended focused assessment with sonography in trauma

Paper	Round Two Votes for Top Five (No (%))	Round Three Votes for Top Five (No (%))	Round Three Total Score	Final Rank
Blaivas M et al. A prospective comparison of supine chest radiography and bedside ultrasound for the diagnosis of traumatic pneumothorax. Acad Emerg Med 2005;12(9):844-9 [23]	8 (57%)	10 (71%)	38	1
Lichtenstein DA, Menu Y. A bedside ultrasound sign ruling out pneumothorax in the critically ill. Lung sliding. Chest 1995;108(5):1345-8 [24]	4 (29%)	9 (64%)	36	2
Lichtenstein D et al. The "lung point": an ultrasound sign specific to pneumothorax. Intensive Care Med 2000;26(10):1434-40 [25]	6 (43%)	9 (64%)	32	3
Chan KK et al. Chest ultrasonography versus supine chest radiography for diagnosis of pneumothorax in trauma patients in the emergency department. Cochrane Database Syst Rev 2020;7(7):CD013031 [26]	6 (43%)	8 (57%)	23	4
Wilkerson RG, Stone MB. Sensitivity of bedside ultrasound and supine anteroposterior chest radiographs for the identification of pneumothorax after blunt trauma. Acad Emerg Med 2010;17(1):11-7 [27]	3 (21%)	5 (36%)	14	5
Akoglu H. Diagnostic accuracy of the Extended Focused Abdominal Sonography for Trauma (E-FAST) performed by emergency physicians compared to CT. Am J Emerg Med 2018;36(6):1014-1017 [28]	5 (36%)	4 (29%)	13	
Melniker LA et al. Randomized controlled clinical trial of point-of-care, limited ultrasonography for trauma in the emergency department: the first sonography outcomes assessment program trial. Ann Emerg Med 2006;48(3):227-35 [11]	4 (29%)	3 (21%)	12	
Stengel D et al. Point-of-care ultrasonography for diagnosing thoracoabdominal injuries in patients with blunt trauma. Cochrane Database Syst Rev 2018;12(12):CD012669 [15]	4 (29%)	6 (43%)	12	
Kirkpatrick AW et al. Hand-held thoracic sonography for detecting post-traumatic pneumothoraces: the Extended Focused Assessment with Sonography for Trauma (EFAST). J Trauma 2004;57(2):288-95 [29]	3 (21%)	3 (21%)	9	
Netherton S et al. Diagnostic accuracy of eFAST in the trauma patient: a systematic review and meta-analysis. CJEM 2019;21(6):727-738 [17]	3 (21%)	3 (21%)	7	
Staub LJ. Chest ultrasonography for the emergency diagnosis of traumatic pneumothorax and haemothorax: A systematic review and meta-analysis. Injury 2018;49(3):457-466 [30]	3 (21%)	5 (36%)	6	
Atkinson P. The V-line: a sonographic aid for the confirmation of pleural fluid. Crit Ultrasound J 2012;4(1):19 [31]	3 (21%)	2 (14%)	4	
Helland G. Comparison of Four Views to Single-view Ultrasound Protocols to Identify Clinically Significant Pneumothorax. Acad Emerg Med 2016;23(10):1170-1175 [32]	3 (21%)	3 (21%)	4	

## Discussion

Summaries of the top five papers for FAST and E-FAST are provided below.

FAST

1. Melniker LA et al. Randomized controlled clinical trial of point-of-care, limited ultrasonography for trauma in the emergency department: the first sonography outcomes assessment program trial. Ann Emerg Med 2006;48(3):227-35 [11].

This randomized controlled study assessed the impact of POCUS compared to usual care in patients with torso trauma and the impact it had on time to operative intervention. Adult and pediatric patients with blunt or penetrating trauma were eligible for enrollment at two trauma centers. Patients were excluded if consent could not be obtained or if immediate transfer to the operating room was required. POCUS was performed by individuals who were trained and credentialed in accordance with the ACEP ultrasound guidelines. Of 444 patients eligible for enrollment, 262 were enrolled and randomized, and ultimately 217 patients completed the trial and were included in the final analysis: 111 in the POCUS group and 106 in the control group. Demographic and injury severity scores between the two groups were comparable. Of these 217 patients, 63 (29%) underwent operative intervention. The primary outcome of time from ED arrival to transfer to operative care was 64% less in the POCUS group compared to the standard care group (57 mins vs 166 mins). Secondary findings demonstrated that patients in the POCUS arm underwent less CT imaging, had shorter hospital length of stay, fewer complications, and 35% less hospital charges compared to the standard care group.

2. Inaba K et al. FAST ultrasound examination as a predictor of outcomes after resuscitative thoracotomy: a prospective evaluation. Ann Surg 2015;262(3):512-8 [12].

This prospective observational study assessed the ability of cardiac POCUS to discriminate survivors and potential organ donors from those who would not survive resuscitative thoracotomy (RT) among patients presenting in traumatic cardiac arrest. The authors enrolled patients presenting to their academic level 1 trauma center in traumatic arrest who received RT in the ED and also underwent cardiac POCUS. Patients undergoing thoracotomy in the operating room were excluded. Cardiac POCUS scans were performed by EM residents who captured parasternal and subxiphoid views under direct faculty supervision to assess for the presence or absence of pericardial effusion and cardiac motion. Over 3.7 years, 187 patients arrived in traumatic arrest, underwent ED RT, and also underwent cardiac POCUS. Of this cohort, six (3.2%) survived and three (1.6%) became organ donors. POCUS demonstrated cardiac motion in 54 (29%) patients, pericardial fluid in 16 patients (9%), and was inadequate in seven individuals (4%). Cardiac motion on POCUS had a sensitivity of 1.00 and specificity of 0.74 for the identification of survivors and organ donors. This study demonstrates that patients who did not have cardiac motion or pericardial effusion on initial cardiac POCUS had a survival rate of zero. POCUS effectively identified those patients who had the potential to survive ED RT from those who did not.

3. Ma OJ et al. Prospective analysis of a rapid trauma ultrasound examination performed by emergency physicians. J Trauma 1995;38(6):879-85 [13].

Early FAST studies were mainly performed by trauma surgeons whereas this prospective study was one of the first to evaluate the accuracy of rapid trauma ultrasonography when performed by emergency physicians. The authors enrolled a convenience sample of adult patients with major blunt or penetrating torso trauma presenting to a level one trauma center in the USA. Patients were excluded if <18 years old or if sonography would delay a procedure or the operating room. The sonographers were nine EM faculty, fellows, and residents who underwent 10 hours of training and a minimum of 15 videotaped scans. Their protocol included views of the right upper quadrant, left upper quadrant, pelvis, and subxiphoid cardiac. Overall, they enrolled 245 patients of which 165 (67%) sustained blunt trauma. There were 32 true positive findings of intraperitoneal free fluid detected by ultrasound with a sensitivity of 0.86, specificity of 0.99, and overall accuracy of 0.98. For pericardial fluid, ultrasonography detected six true positive cases with an overall sensitivity of 1.00, specificity of 0.99, and accuracy of 0.99. The overall sensitivity and specificity of ultrasound to detect free fluid in the intracavitary spaces was similar for penetrating and blunt trauma.

4. Plummer D et al. Emergency department echocardiography improves outcome in penetrating cardiac injury. Ann Emerg Med 1992;21(6):709-12 [14].

This retrospective study evaluated the impact of emergency echocardiography in patients with penetrating cardiac injuries presenting to a large urban trauma center over a 10-year period. Emergency echocardiography was being introduced into their ED at that time. They included all patients with confirmed cardiac or great vessel injury, and compared those who received emergency echocardiography with those who did not. They identified 49 patients, with 28 in the echocardiography group and 21 in the non-echocardiography group. Although the groups had similar injury severity, there was a significantly higher rate of survival for the echocardiography group compared with the non-echocardiography group (100% vs 57%, p=0.003). The echocardiography group had improved neurologic outcome (p=0.008) and faster time to the operating room than the non-echocardiography group (16 mins vs 43 mins, p<0.001). Emergency echocardiography had the highest impact for patients with severe life-threatening injuries, where the rates of survival were 100% in the echocardiography group and only 31% in the non-echocardiography group.

5. Stengel D et al. Point-of-care ultrasonography for diagnosing thoracoabdominal injuries in patients with blunt trauma. Cochrane Database Syst Rev 2018;12(12):CD012669 [15].

This was a systematic Cochrane review aimed at determining the diagnostic accuracy of POCUS for detecting and excluding free fluid, organ injuries, vascular lesions and other injuries compared to a diagnostic reference standard (CT, MRI, laparotomy, thoracotomy, and autopsy) in patients with blunt thoracoabdominal trauma. A total of 34 studies with 8,635 participants were included. Overall, the studies were quite heterogeneous. The summary estimate of sensitivity and specificity was 0.74 (CI 95%, 0.65-0.81) and 0.96 (CI 95%, 0.94-0.98). The overall positive likelihood ratio was 18.5 (10.8-40.5) and the negative likelihood ratio was 0.27 (0.19-0.37). The reported accuracy of POCUS depended on the body area being examined. For abdominal trauma, the sensitivity and specificity of POCUS for diagnosing intra-abdominal injury was 0.68 (95% CI 0.59-0.75) and 0.95 (95% CI 0.92-0.97). For chest trauma, the accuracy for detection of pneumothorax in adult patients was impressive, with a sensitivity of 0.96 (95% CI 0.88-0.99) and a specificity of 0.99 (95% CI 0.97-1.00). Based on these results, the authors recommend including POCUS as part of trauma resuscitation for adult patients given its high specificity. They also recommend confirmatory testing be considered in patients with high probability of thoracoabdominal injury and negative POCUS findings given the high risk of a false negative scan.

E-FAST

1. Blaivas M et al. A prospective comparison of supine chest radiography and bedside ultrasound for the diagnosis of traumatic pneumothorax. Acad Emerg Med 2005;12(9):844-9 [23].

This prospective, single-blinded study enrolled a convenience sample of 176 level one trauma patients. All patients received both supine portable chest x-ray (CXR) and POCUS immediately followed by a CT chest as the gold standard. POCUS images were obtained and interpreted by one of five emergency physicians, while the CXR was interpreted by the attending trauma physician. Both CXR and POCUS were compared to a reference standard of either CT chest or air release on chest tube placement if placed prior to CT. Lung sliding on B mode, or power doppler in obscured views, were considered negative findings, and the absence of lung sliding on both was considered positive. Of the study population, 53 (30%) patients had pneumothorax, with 12 undergoing chest tube placement prior to CT. CXR identified 40 of these patients to yield a sensitivity and specificity of 0.75 (95% CI 0.62-0.86) and 1.00 (95% CI 0.97-1.00). POCUS identified 53 pneumothoraces, for a sensitivity of 0.98 (95% CI 0.90-1.00) and specificity of 0.99 (95% CI 0.96-1.00). The POCUS arm had one false positive (large lung contusion) and one false negative (1% pneumothorax). The Spearman’s rank correlation between CT and POCUS was very strong at 0.82. Assessment of pneumothorax size on POCUS correlated best with either small or large pneumothoraces.

2. Lichtenstein DA, Menu Y. A bedside ultrasound sign ruling out pneumothorax in the critically ill. Lung sliding. Chest 1995;108(5):1345-8 [24].

Norman Rantanen, a veterinarian, provided the first sonographic description of pneumothorax in 1986, detailing the disappearance of an ultrasound respiratory movement at the lung surface [33]. Daniel Lichtenstein subsequently coined the term “lung sliding” to describe this ultrasound respiratory movement. He defined “lung sliding” as a to-and-fro movement of the pleural line synchronized with respiration. In this 1995 study, he was the first to evaluate the test characteristics of “lung sliding” for diagnosing pneumothorax in critically ill, ventilated patients compared to a reference standard of CT. The authors sonographically interrogated the anterior chest wall of 111 hemi-thoraces, of which there were 43 cases of pneumothorax. Of these 43 cases of pneumothorax, two could not be analyzed due to subcutaneous emphysema, yielding a 98% feasibility rate for the ultrasound examination. The presence of “lung sliding” excluded pneumothorax in these patients with a negative predictive value of 1.00; 62 patients had “lung sliding” and none had pneumothorax on CT. In this series, the sensitivity of loss of “lung sliding” to diagnose pneumothorax was 0.95 and specificity was 0.91. Ultrasound visualization of “lung sliding” is correlated with the absence of pneumothorax, but the loss of “lung sliding” was suggestive but not sufficient to confirm pneumothorax.

3. Lichtenstein D et al. The "lung point": an ultrasound sign specific to pneumothorax. Intensive Care Med 2000;26(10):1434-40 [25].

Daniel Lichtenstein introduced the concept of the “lung point” to the ultrasound literature in this ICU-based study. When using ultrasound to exclude pneumothorax, the presence of lung sliding or comet-tail artifacts reliably excludes this diagnosis. The absence of lung sliding is suggestive of pneumothorax but is not perfectly specific. Lichtenstein defined this novel “lung point” sign as a dynamic change in sonographic appearance from suggesting pneumothorax (absence of lung sliding with A lines) to either lung sliding, B lines, or an alteration of the visualized A lines. This change in pattern should appear on inspiration and disappear on expiration with the probe held in the same location throughout. A blinded, expert operator performed lung ultrasound on 70 CT-confirmed pneumothoraces in 64 patients, as well as a control group of 238 hemi-thoraces from 119 patients. In the study group, 66 of 70 bedside ultrasounds were interpretable as subcutaneous emphysema prevented sonographic analysis in four cases. The “lung point” was visualized in 44 of 66 pneumothoraces. In the control group, 236 of 238 bedside ultrasounds were feasible; one patient had a large dressing preventing assessment and one patient had calcified pleura making the scan uninterpretable. Of this control group, none of these patients had a “lung point”. Overall, the examination had a 98% feasibility rate, and the “lung point” sign had a sensitivity of 0.66 and specificity of 1.00 for pneumothorax.

4. Chan KK et al. Chest ultrasonography versus supine chest radiography for diagnosis of pneumothorax in trauma patients in the emergency department. Cochrane Database Syst Rev 2020;7(7):CD013031 [26].

This high-quality systematic review from the Cochrane Library compared the diagnostic accuracy of chest POCUS by frontline non-radiologist physicians versus supine CXR for the diagnosis of pneumothorax in trauma patients, both blunt and penetrating, in the ED. They identified 13 prospective, paired comparative accuracy studies comparing chest POCUS to supine CXR, and of these, nine studies used patients as the unit of analysis. These nine studies, which included 1,271 patients of whom 410 had traumatic pneumothorax confirmed by a reference standard of chest CT or tube thoracostomy, were used in the primary analysis. There was risk of bias in at least one domain, with substantial heterogeneity in the sensitivity of supine CXR amongst most included studies. Overall, POCUS was significantly more accurate than CXR in diagnosing traumatic pneumothorax, with a summary sensitivity of POCUS of 0.91 (95% CI 0.85-0.94) compared to 0.47 (95% CI 0.31-0.63) for CXR, an absolute difference in sensitivity of 0.44 (95% CI 0.27-0.61, p<0.001). There was no significant difference in specificity of POCUS at 0.99 (95% CI 0.97-1.00) compared with 1.00 (95% CI 0.97-1.00) for CXR. In a hypothetical cohort of 100 patients, where 30 have traumatic pneumothorax, POCUS would miss three cases (false negatives) and over-diagnose only one without pneumothorax (false positive), whereas CXR would miss 16 cases with zero over-diagnoses. This review further confirms the added benefit of chest POCUS as part of the E-FAST.

5. Wilkerson RG, Stone MB. Sensitivity of bedside ultrasound and supine anteroposterior chest radiographs for the identification of pneumothorax after blunt trauma. Acad Emerg Med 2010;17(1):11-7 [27].

This systematic review compared the diagnostic accuracy of POCUS and CXR for pneumothorax secondary to blunt trauma. Specifically, the authors searched for prospective observational trials comparing the diagnostic performance of POCUS by emergency physicians to portable supine anteroposterior CXR during the initial ED evaluation of blunt trauma adult patients ≥18 years old with suspicion of pneumothorax. The criterion standard was chest CT, or for unstable patients, the release of air with chest tube placement. Their search yielded 208 citations, and ultimately four studies including 606 patients were selected for final inclusion. In all the included studies, POCUS demonstrated a higher sensitivity (0.86-0.98) compared to CXR (0.28-0.76%) for the detection of pneumothorax, and POCUS had comparable specificity (0.97-1.00) to CXR (1.00). The overall quality of the studies was limited by convenience sampling, nonrandomized design, exclusion of those in whom the POCUS assessment could not be completed, heterogeneity with operator training, and lack of assessment of interobserver agreement. Of note, the Blaivas et al study [16] was included in this systematic review.

Limitations

Our expert panel consisted of Canadian EM POCUS experts. It is possible that POCUS experts from a different specialty outside of EM, like trauma surgery, may have selected different papers. Due to the panel's composition of entirely POCUS experts, there may be selection bias in favor of papers that are positive for POCUS. Because our method of establishing a top five list assigned weighted points based on how individual experts ranked their top five list, one of the papers selected to the top five E-FAST list had relatively low consensus based on the total number of votes received in round three. Given the constraints of a top five list, there are additional papers of value in tables one and two related to both FAST and E-FAST that may be worth reading. Another curious result of our modified Delphi process is that none of the top five papers for E-FAST specifically studied the use of POCUS for traumatic hemothorax. This may be due to the fact that POCUS identification of traumatic hemothorax is a subcategory of POCUS identification of pleural fluid or effusion, so our protocol may have been too specific with its explicit focus on traumatic hemothorax as a component of the E-FAST.

## Conclusions

We have developed a reading list of the top five influential papers for FAST and E-FAST that will benefit residents, fellows, and clinicians who are interested in the evidence supporting our use of POCUS. It is specifically relevant to EM practitioners, but will be useful to clinicians in all specialties who want to implement the use of POCUS into their practice in an evidence-informed manner. Future iterations of this process should generate a list of the most influential POCUS papers published for other major applications and uses of POCUS.

## Appendices

**Table 3:** Expert panel members listed in alphabetical order and their academic affiliations

Expert Panel Member	Academic University Affiliation
Paul Atkinson	Dalhousie University
Colin Bell	University of Calgary
Talia Burwash-Brennan	Université de Montréal
Ian Buchanan	McMaster University
Jordan Chenkin	University of Toronto
Claire Heslop	University of Toronto
Tom Jelic	University of Manitoba
Daniel Kim	University of British Columbia
Elizabeth Lalande	Université Laval
David Lewis	Dalhousie University
Frank Myslik	Western University
Laurie Robichaud	McGill University
Gillian Sheppard	Memorial University of Newfoundland
Rajiv Thavanathan	University of Ottawa
